# Evidence of Site‐Specific Mucosal Autoantibody Secretion in Rheumatoid Arthritis

**DOI:** 10.1002/art.43036

**Published:** 2024-11-18

**Authors:** Veerle F. A. M. Derksen, Klara Martinsson, Anouk G. van Mourik, Carlijn A. Wagenaar, René E. M. Toes, Wendy Walrabenstein, Daniel Sjöberg, Dirkjan van Schaardenburg, Tom W. J. Huizinga, Alf Kastbom, Anna Svärd, Diane van der Woude

**Affiliations:** ^1^ Leiden University Medical Center Leiden The Netherlands; ^2^ Linköping University Linköping Sweden; ^3^ Reade and Amsterdam University Medical Center Amsterdam The Netherlands; ^4^ Amsterdam Rheumatology and Immunology Center Reade Amsterdam The Netherlands; ^5^ Uppsala University, Uppsala, Sweden, and Falun Hospital Falun Sweden; ^6^ Linköping University, Linköping, Sweden, and Uppsala University Uppsala Sweden

## Abstract

**Objective:**

Anti–citrullinated protein antibodies (ACPA) have been detected in sputum and saliva, indicating that anti–modified protein antibodies (AMPA) can be produced at mucosal sites in patients with rheumatoid arthritis (RA). However, the body's largest mucosal compartment, the gut, has not yet been examined. We therefore investigated the presence of several AMPA (ACPA, anti–carbamylated protein antibodies [anti‐CarP], and anti–acetylated protein antibodies [AAPA]) at different mucosal sites, including the intestinal tract.

**Methods:**

Paired fecal/ileal wash, saliva, and serum samples of patients with RA and healthy volunteers were collected in two independent cohorts. Data involving feces were replicated in a third cohort. In these secretions, AMPA were analyzed using in‐house enzyme‐linked immunosorbent assay with unmodified peptides as control. In fecal samples, total IgA and anti–*Escherichia coli* IgA were measured.

**Results:**

ACPA, anti‐CarP, and AAPA IgA were measurable in saliva of seropositive patients with RA (prevalence 9%–40%). No AMPA could be detected in feces. IgA was present because total IgA and anti–*E. coli* IgA were detectable in feces of ACPA‐positive patients with RA and healthy donors. Results were confirmed in another cohort using colonoscopically collected ileal wash samples.

**Conclusion:**

Our study shows the presence of ACPA, anti‐CarP, and AAPA IgA in saliva of ACPA‐seropositive patients with RA. However, no AMPA could be detected in feces/ileal wash samples of these patients, although our assays were able to measure other antigen‐specific antibodies. These data suggest that mucosal autoantibody secretion may occur in the oral mucosa of patients with RA, whereas no evidence could be found for this process in the lower gastrointestinal tract.

## INTRODUCTION

The presence of anti–modified protein antibodies (AMPA) directed against post‐translational modified proteins (PTMs) is a hallmark of seropositive rheumatoid arthritis (RA). The most well‐known and clinically important AMPA are anti–citrullinated protein antibodies (ACPA), whereas other AMPA recognize anti–carbamylated protein antibodies (anti‐CarP) or anti–acetylated protein antibodies (AAPA). The processes leading to the break of tolerance against PTMs, to the maturation of the AMPA response, and eventually to the development of seropositive RA are not fully understood. One of the hypotheses gaining increased attention is that mucosal surfaces play a role in AMPA formation.^1^, ^2^, ^3^ Inflammation at mucosal surfaces, triggered by environmental factors and microbiome–host interactions in combination with the local presence of PTMs, might create conditions in which tolerance to PTMs is broken.^1^, ^2^, ^4^, ^5^

There is accumulating evidence that the airway mucosa is involved in seropositive RA. Smoking, along with exposure to silica dust and other inhalants, is a major risk factor for the development of seropositive RA and the concurrent presence of multiple RA‐associated antibodies.^6^, ^7^, ^8^ It is hypothesized that these environmental factors induce airway inflammation, which can contribute to autoantibody formation.^9^ However, the lungs are not the only mucosal sites that may be involved in RA development. The epidemiologic association between RA and periodontitis was already reported years ago.^10^ Oral and intestinal microbiome disturbances have been described in patients with RA and people at risk of developing RA.^11^, ^12^, ^13^, ^14^ Novel research shows that patients with RA with ongoing periodontitis experience repeated bacteremia with oral bacteria. Bacteria can be citrullinated, and citrullinated bacterial epitopes can be recognized by ACPAs. These findings provide an interesting potential link between physiologic antibacterial responses and autoimmunity in RA.^15^ The association between the lung and oral mucosa and RA is further substantiated by the discovery that ACPA can be present in sputum, bronchoalveolar fluid, and saliva of patients with RA.^16^, ^17^, ^18^ Rheumatoid factor can also be present in the saliva of those patients.^19^

However, the largest mucosal site in the body, the intestine, has received less attention over the years compared to the lung and mouth. Lately, this has changed with the finding that monoclonals derived from circulating plasmablasts in individuals at risk of RA can bind both RA‐associated citrullinated autoantigens and bacteria in feces.^20^ These findings suggest that the intestinal mucosa might also be involved in the pathophysiology of RA. The presence of other AMPA (besides ACPA) at mucosal sites has not yet been investigated, although this could provide new information on the development of the AMPA response in RA, especially in the gut. AAPA might provide an interesting angle when it comes to mucosal microbial exposures as a potential trigger for autoimmunity because various bacterial species use acetylation of self‐proteins to regulate cell processes.^21^, ^22^ Anti‐CarP responses may also have an intestinal origin because carbamylation has been shown to occur in the human gastrointestinal tract.^23^ It therefore appears plausible that anti‐CarP and AAPA could be produced at mucosal sites and that local availability of specific post‐translational modifications, as a product of microbiome, food constituents, and host cell interactions, might diversify and broaden the AMPA response in RA. Nevertheless, whether AMPAs are secreted in the intestinal tract is currently unknown.

Hence, we investigated whether ACPA, anti‐CarP, and AAPA can all be detected in mucosal secretions, with emphasis on material derived from the intestinal tract of patients with RA. To this end, we collected paired serum, saliva, and feces of patients with RA and healthy donors and tested these samples for the presence of ACPA, anti‐CarP, and AAPA. Two other independent cohorts were used to replicate our findings.

## PATIENTS AND METHODS

### Cohorts

In the Dutch MUCosal Origin of Serum Autoantibodies in rheumatoid arthritis (MUCOSA) study, paired serum, saliva, and feces samples were collected cross‐sectionally from 47 patients with RA visiting the outpatient clinic (of whom 36 were ACPA seropositive) and from 21 healthy controls. Saliva was collected by passive drooling, and feces were collected by participants themselves and immediately frozen. One patient had severe hyposalivation precluding the collection of saliva. Details are provided in Supplementary Data 1.

To substantiate our findings, we made use of samples from another independent study. The Swedish IntestRA study included 20 ACPA‐seropositive patients with RA, 10 healthy donors, and 9 patients with inflammatory bowel disease (IBD) as additional controls. Serum and saliva samples were collected in a similar method. However, to investigate the presence of autoantibodies in the gut, ileal wash fluid was collected via colonoscopy instead. Details are provided in Supplementary Data 2.

Feces samples from a third cohort, the Dutch Plants for Joints (PFJ) trial,^24^, ^25^ were examined to corroborate the results regarding AMPA in feces. Baseline feces samples of 42 ACPA‐seropositive patients with RA and 10 patients with osteoarthritis as control were investigated. Feces were collected by participants themselves at home and sent by mail, after which samples were stored at −80°C.

In all three studies, all patients fulfilled the 2010 American College of Rheumatology/EULAR criteria for RA, and most had longstanding disease. Throughout this manuscript, ACPA seropositive refers to ACPA IgG seropositivity. All studies were performed in concordance with the Declaration of Helsinki, approved by the relevant local medical ethical committees, and all participants provided written informed consent.

### Measurements

The presence of AMPAs and rheumatoid factor (RF) in serum, saliva, and feces in the MUCOSA study was tested by in‐house enzyme‐linked immunosorbent assay (ELISA) using peptides containing different modifications on a Cyclic Citrullinated Peptide 2 backbone. Measurements in the PFJ trial were performed in accordance with the protocols used for feces samples in the MUCOSA study. In the IntestRA study, modified commercial anti‐CCP assays (CCPlus Immunoscan, Svar Life Science) were used to measure ACPA IgA in ileal wash and saliva samples. Autoantibody analyses in the IntestRA study focused on ACPA only. Total IgA levels and anti–*Escherichia coli* antibodies were also measured in mucosal secretions by ELISA. For anti–*E. coli*, ELISA plates were coated with *E. coli* lysates. After blocking and adding undiluted fecal extracts, horseradish peroxidase‐labeled detection antibodies were used stepwise before visualization with ABTS (Supplementary Data 1). Furthermore, analysis of markers of inflammation in saliva (total protein and matrix metalloproteinase‐8 [MMP‐8] levels) and feces (calprotectin) was performed in the MUCOSA study using ELISA kits (Total MMP‐8 ELISA kit, R&D systems, DMP800B; and Calprotectin ELISA kit, Orgentec, ORG580) according to manufacturer's instruction.

Saliva samples were homogenized and centrifugated before use to remove any debris. To be able to detect autoantibodies in fecal matter, protein fractions were prepared by diluting the feces 1:5 in feces dilution buffer (phosphate‐buffered saline + 0.05 M ethylenediaminetetraacetic acid + 1.66 mM phenylmethylsulfonyl fluoride + 0.1 mg/mL soybean trypsin inhibitor [Sigma]). Samples were mixed vigorously for 10 to 20 minutes until homogeneous and spun down. Ileal wash samples were centrifuged and frozen within one hour after collection.

When testing for AMPA positivity, all samples were also measured simultaneously on the unmodified control peptide to investigate whether binding was specific for the post‐translational modification. In the MUCOSA study and PFJ trial, a sample was considered AMPA positive when both of the following criteria were met: 1) The value measured on the modified peptide was higher than the cut‐off based on the mean plus two times the SD of the signal of healthy controls on that peptide, and 2) the optical density (OD) of the signal measured on the modified peptide was >2 times higher than the OD of the same sample measured on the unmodified peptide. In the IntestRA study, OD signals on the arginine control were first subtracted from the ACPA OD values. Thereafter, a cut‐off was calculated in a similar manner.

More information about sample processing and autoantibody detection can be found in Supplementary Data 1 (MUCOSA and PFJ) and 2 (IntestRA). Mann‐Whitney U tests, chi‐square tests, or Fisher's exact tests, as appropriate depending on the kind of data, were performed to compare antibody positivity and markers of inflammation among groups.

## RESULTS

### AMPAs in saliva

**Figure 1:**
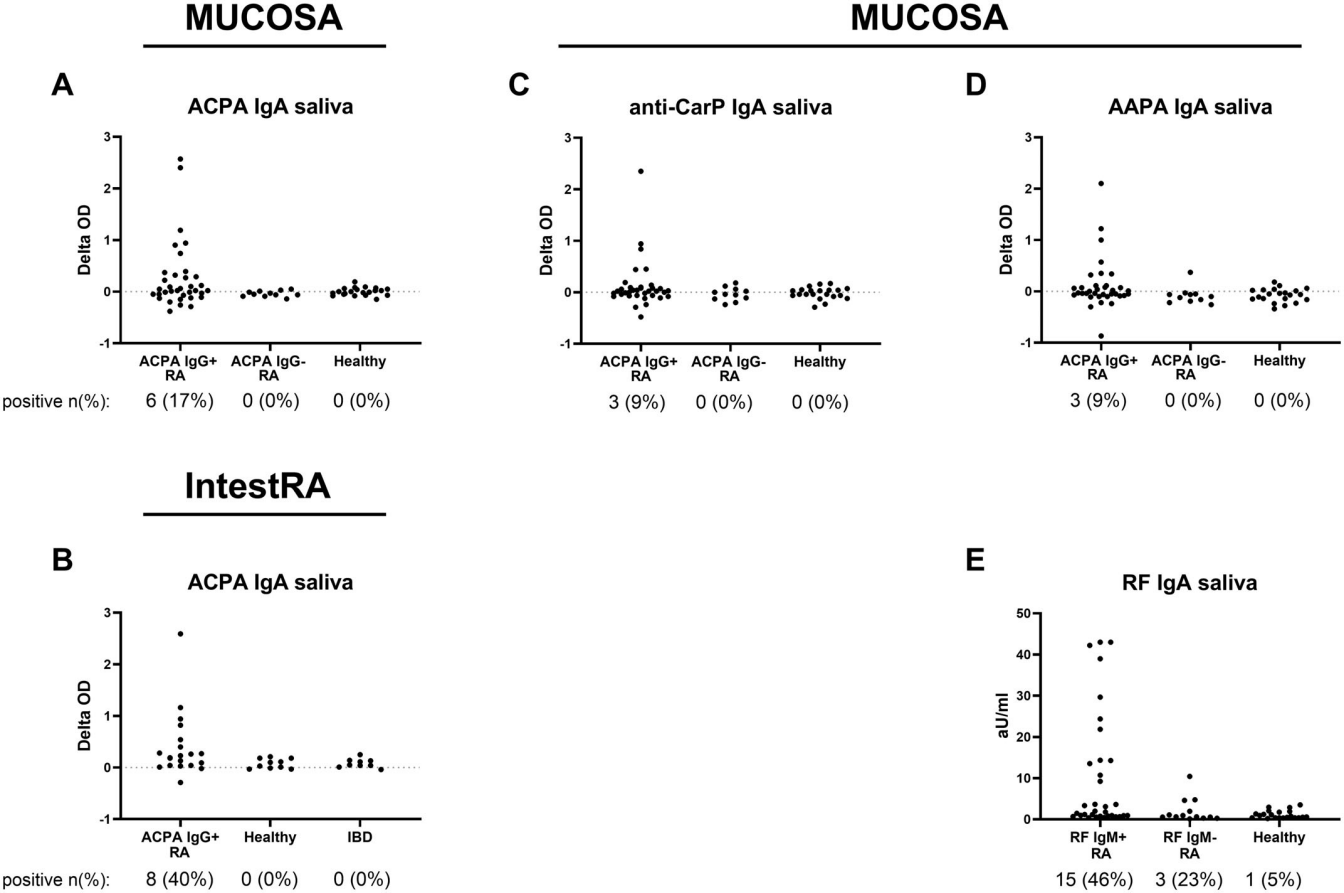
Autoantibody measurements in saliva. (A) ACPA IgA in saliva in the MUCOSA study, (B) ACPA IgA in saliva in the IntestRA study, and (C) anti‐CarP IgA, (D) AAPA IgA, and (E) RF IgA in saliva in the MUCOSA study are shown. Delta OD (difference in OD between modified peptide and unmodified peptide) for AMPAs and aU/mL (arbitrary units per milliliter) for RF are depicted. Groups on *x* axis are based on diagnosis and seropositivity (panel E uses RF IgM seropositivity to define groups). The number (%) of positive patients for that specific autoantibody is given. AAPA, anti–acetylated protein antibody; ACPA, anti–citrullinated protein antibody; AMPA, anti–modified protein antibody; CarP, carbamylated protein; IBD, inflammatory bowel disease; MUCOSA, MUCosal Origin of Serum Autoantibodies in rheumatoid arthritis; OD, optical density; RA, rheumatoid arthritis; RF, rheumatoid factor.

Mucosal autoantibodies were investigated in three independent cohorts, of which the clinical characteristics are listed in Table 1. First, autoantibodies were measured in serum and saliva of participants in the MUCOSA study. Seventeen percent of ACPA‐seropositive patients with RA in the MUCOSA study had detectable ACPA IgA in saliva, whereas ACPA could not be detected in the saliva of patients who were ACPA seronegative or healthy donors (Figure 1A) (not significant [ns]). In the Swedish IntestRA study, ACPA IgA was found in saliva of 40% of the patients who were ACPA seropositive (Figure 1B), whereas none of the patients with IBD or healthy controls were positive (*P* = 0.03 compared with healthy controls). However, the presence of AMPA was not limited to ACPA IgA. Also, anti‐CarP IgA and AAPA IgA could be detected in saliva of seropositive patients with RA in the MUCOSA study (Figure 1C and D), although the number of patients positive for salivary autoantibodies was low (9%) for both autoantibodies. RF IgA could be found in saliva 46% of ACPA‐seropositive patients with RA (Figure 1E) (*P* = 0.001 compared with healthy controls) and was present more frequently compared with salivary ACPA IgA.

**Table 1:** Patient characteristics of all cohorts*

Characteristics	MUCOSA		IntestRA			Plants for Joints	
	RA, n = 47	Healthy, n = 21	RA, n = 20	Healthy, n = 10	IBD, n = 9	RA, n = 42	OA, n = 10
Age, y, mean ± SD	59 ± 13	48 ± 16	61 ± 9	62 ± 9	30 ± 8	55 ± 12	62 ± 6
Female, n (%)	37 (79)	13 (62)	15 (75)	7 (70)	6 (67)	36 (86)	10 (100)
Disease duration, years, median (IQR)	14 (8–16)	–	0.7 (0–12)	–	–	6.5 (3–15)	–
Smoking ever, n (%)	26 (55)	6 (29)	12 (60)	4 (44) (n = 9)	3 (33)	–	–
ESR, mm/h, median (IQR)	9 (2–34)	–	13 (8–20)	6 (4–24)	8 (4–15)	15 (8–32)	–
DAS28, median (IQR)	2.6 (1.7–3.6) (n = 45)	–	3.0 (2.0–4.0) (n = 10)	–	–	3.9 (3.2–4.5)	–
Serum antibody positivity^a^							
ACPA IgG, n (%)	36 (77)	0 (0)	20 (100)	0 (0)	0 (0)	42 (100)	–
Anti‐CarP IgG, n (%)	21 (45)	0 (0)	–	–	–	–	–
AAPA IgG, n (%)	21 (45)	0 (0)	–	–	–	–	–
ACPA IgA, n (%)	18 (38)	0 (0)	20 (100)	0 (0)	0 (0)	–	–
Anti‐CarP IgA, n (%)	4 (9)	0 (0)	–	–	–	–	–
AAPA IgA, n (%)	5 (11)	0 (0)	–	–	–	–	–
RF IgM n (%)	34 (72)	3 (14)	–	–	–	–	–
RF IgA, n (%)	21 (45)	1 (5)	–	–	–	–	–

* Characteristics of all cohorts at inclusion. AAPA, anti–acetylated protein antibodies; ACPA, anti–citrullinated protein antibodies; CarP, carbamylated protein antibodies; DAS28, Disease Activity Score in 28 joints; ELISA, enzyme‐linked immunosorbent assay; ESR, erythrocyte sedimentation rate; IBD, inflammatory bowel disease; IQR, interquartile range; OA, osteoarthritis; RA, rheumatoid arthritis; RF, rheumatoid factor.

^a^ All serum autoantibody measurements in the MUCOSA study are done with in‐house ELISAs.

**Figure 2:**
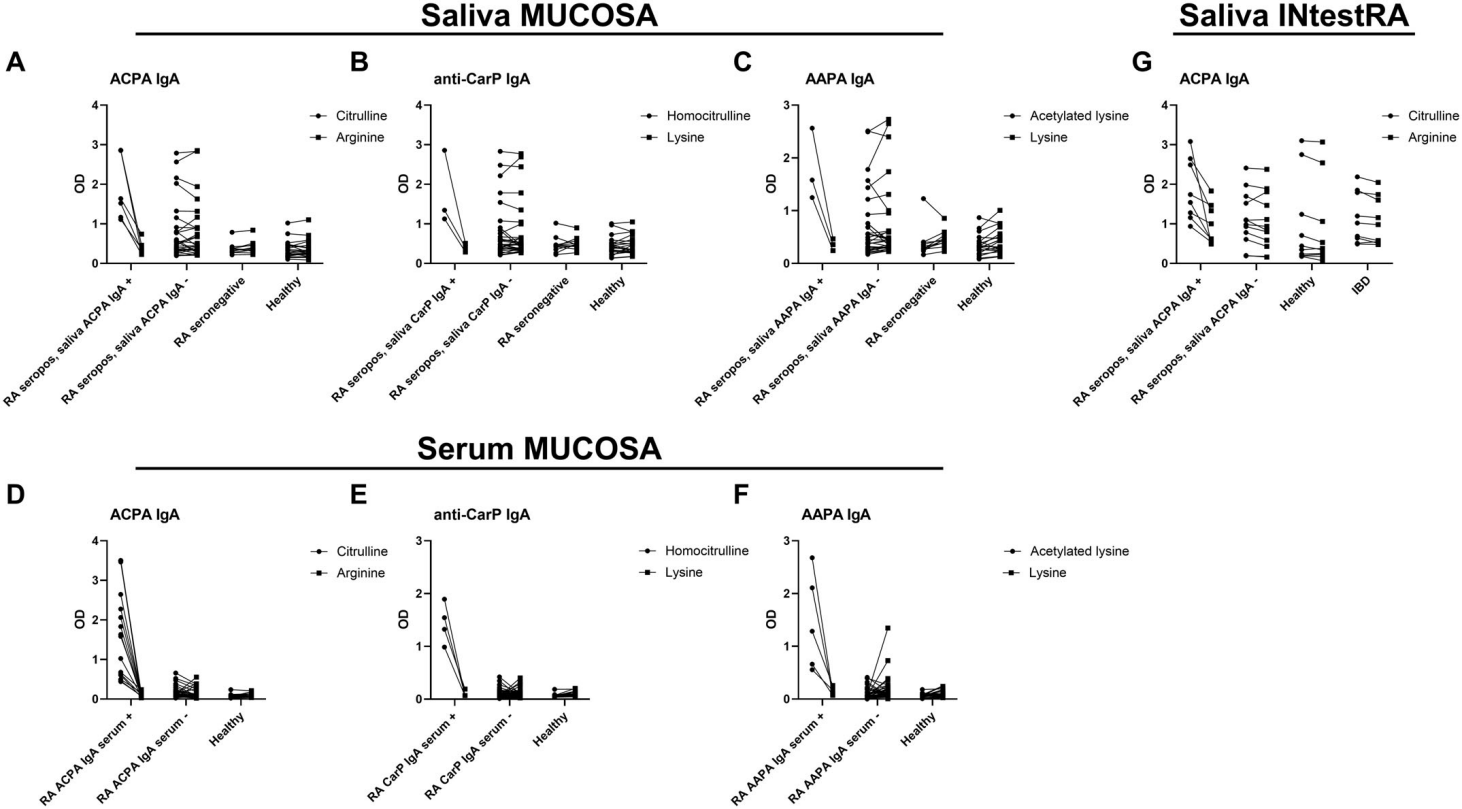
Paired OD values on the modified and unmodified peptide for each AMPA in saliva (A–C) and serum (D–F) of patients and healthy donors in the MUCOSA study and in saliva (G) of patients and controls in the IntestRA study. (A–C) First group of each graph shows all seropositive patients with RA who tested positive for that specific AMPA in saliva, and the second group depicts all seropositive patients with RA who tested negative for that specific AMPA in saliva. (D–F) First group shows patients who were positive for that specific AMPA IgA in serum, and the second group shows patients negative for that specific AMPA IgA in serum. AAPA, anti–acetylated protein antibody; ACPA, anti–citrullinated protein antibody; AMPA, anti–modified protein antibody; CarP, carbamylated protein; IBD, inflammatory bowel disorder; MUCOSA, MUCosal Origin of Serum Autoantibodies in rheumatoid arthritis; OD, optical density; RA, rheumatoid arthritis.

Notably, when examining reactivity to the modified peptides (citrulline, homocitrulline, and acetylated lysine) and unmodified peptides (arginine and lysine, respectively) in saliva in more detail, a substantial number of seropositive patients with RA, but not seronegative patients or healthy controls, had high reactivity to the modified peptide but also showed a similarly high degree of reactivity to the unmodified peptide (Figure 2A–C). When the reactivity to the modified and unmodified peptide was similar, antibody binding was not specific for the PTM, and samples were considered AMPA negative. The high signal measured on the unmodified peptide in saliva samples is markedly different from serum, where background signals are usually very low (Figure 2D–F). In the IntestRA study, unmodified peptide signals were more equally distributed between patients with RA and controls (Figure 2G) (ns).

Next, we investigated whether the presence of autoantibodies in saliva was related to the amount of total IgA in these samples, as salivary IgA levels can differ among individuals and over time.^27^ There was no significant difference in salivary total IgA between seropositive patients with RA who were positive for AMPA in their saliva and those who were negative (Supplementary Figure 1). Therefore, it seems that prevalence of IgA AMPA in saliva cannot solely be attributed to differences in salivary total IgA levels, but may rather point to inherent differences among patients.

### AMPA profile in saliva and serum

**Figure 3:**
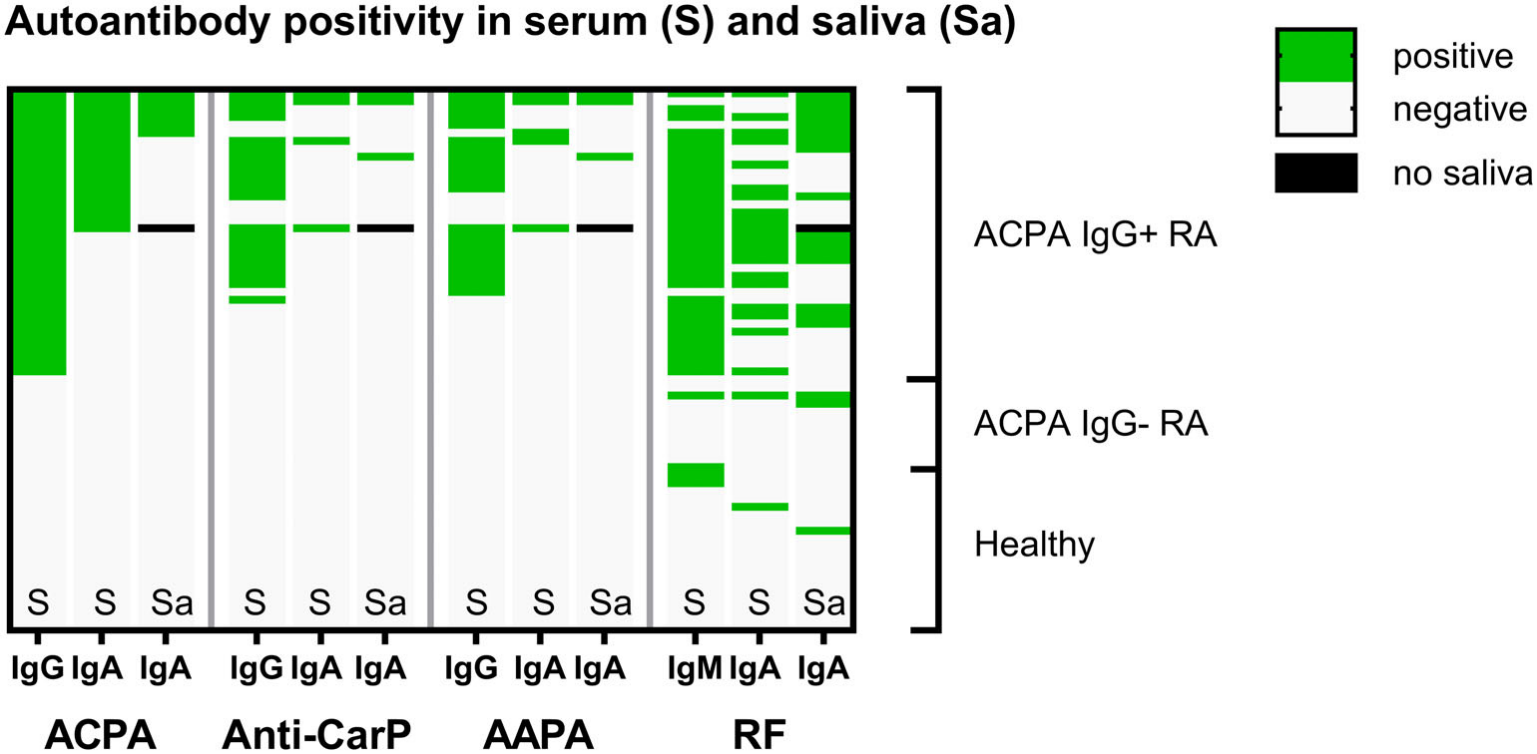
Autoantibody profile in serum (S) and saliva (Sa) in the MUCOSA study. Positivity for autoantibodies in serum and saliva is shown. Each row depicts a study participant. Black field indicates no saliva available due to hyposalivation. AAPA, anti–acetylated protein antibody; ACPA, anti–citrullinated protein antibody; CarP, carbamylated protein; MUCOSA, MUCosal Origin of Serum Autoantibodies in rheumatoid arthritis; RA, rheumatoid arthritis; RF, rheumatoid factor.

Different types of AMPA in saliva tend to co‐occur. Among the seven patients with AMPA‐positive saliva in the MUCOSA study, two were triple positive for ACPA, anti‐CarP, and AAPA, and one patient was anti‐CarP and AAPA double positive (Figure 3). Furthermore, six patients with AMPA‐positive saliva were also saliva RF IgA positive. The presence of an AMPA in saliva always coincided with the presence of that specific AMPA in serum, although the isotype could differ (Figure 3). For example, one patient was positive for AAPA IgA in saliva, whereas AAPA IgG, but no AAPA IgA, could be detected in serum. Similar findings were made for RF. However, three patients with RA who tested positive for RF IgA in saliva tested negative for both RF IgM and IgA in serum. This suggests a local origin and subsequent secretion of autoantibodies rather than leakage from serum antibodies to the saliva. Furthermore, the amount of IgG measured in these saliva samples is, on average ± SD, 9 ± 8 μg/mL, a thousandfold lower than serum IgG (reference levels 7–16 g/L), making contamination of saliva samples with serum autoantibodies in measurable amounts less likely. Thus, the saliva autoantibody profile displays similarity to, but does not necessarily originate from, the serum autoantibody profile.

### Saliva AMPAs and local inflammation

Next, we investigated whether the presence of salivary autoantibodies was associated with oral inflammation. Total protein content, MMP‐8 levels, and total IgA levels in saliva have been determined as markers of local inflammation.^27^, ^28^ In the MUCOSA study, patients with RA in general had slightly lower total IgA levels in saliva compared to healthy donors, whereas there was a nonsignificant trend toward higher MMP‐8 and total protein values in patients with RA. There were no significant differences in all three salivary markers of inflammation between patients with RA who were positive for ACPA in their saliva (median [interquartile range, IQR]: total protein 1,380 μg/mL [1,057–1,747], total IgA 343 μg/mL [253–562], MMP‐8 123 ng/mL [33–150]) and those who were not (median [IQR]: total protein 1,411 μg/mL [1,040–1,621], total IgA 259 μg/mL [169–386], MMP‐8 83 ng/mL [26–138]) (Supplementary Figure 2A–C). Because the number of patients who were ACPA saliva positive is small, salivary markers of inflammation were also compared among ACPA‐seropositive patients with RA, ACPA‐seronegative patients with RA, and healthy donors (Supplementary Figure 2D–F), which showed similar results. Because smoking directly affects the oral mucosa, the relation between autoantibody positivity in saliva and smoking was examined. No significant relation between smoking and the presence of autoantibodies was seen in saliva (Supplementary Table 1), despite the significant association between RF IgM seropositivity and smoking in the MUCOSA study (*P* = 0.04) and a similar trend for ACPA seropositivity (Supplementary Table 1).

### AMPA in the intestinal tract

**Figure 4:**
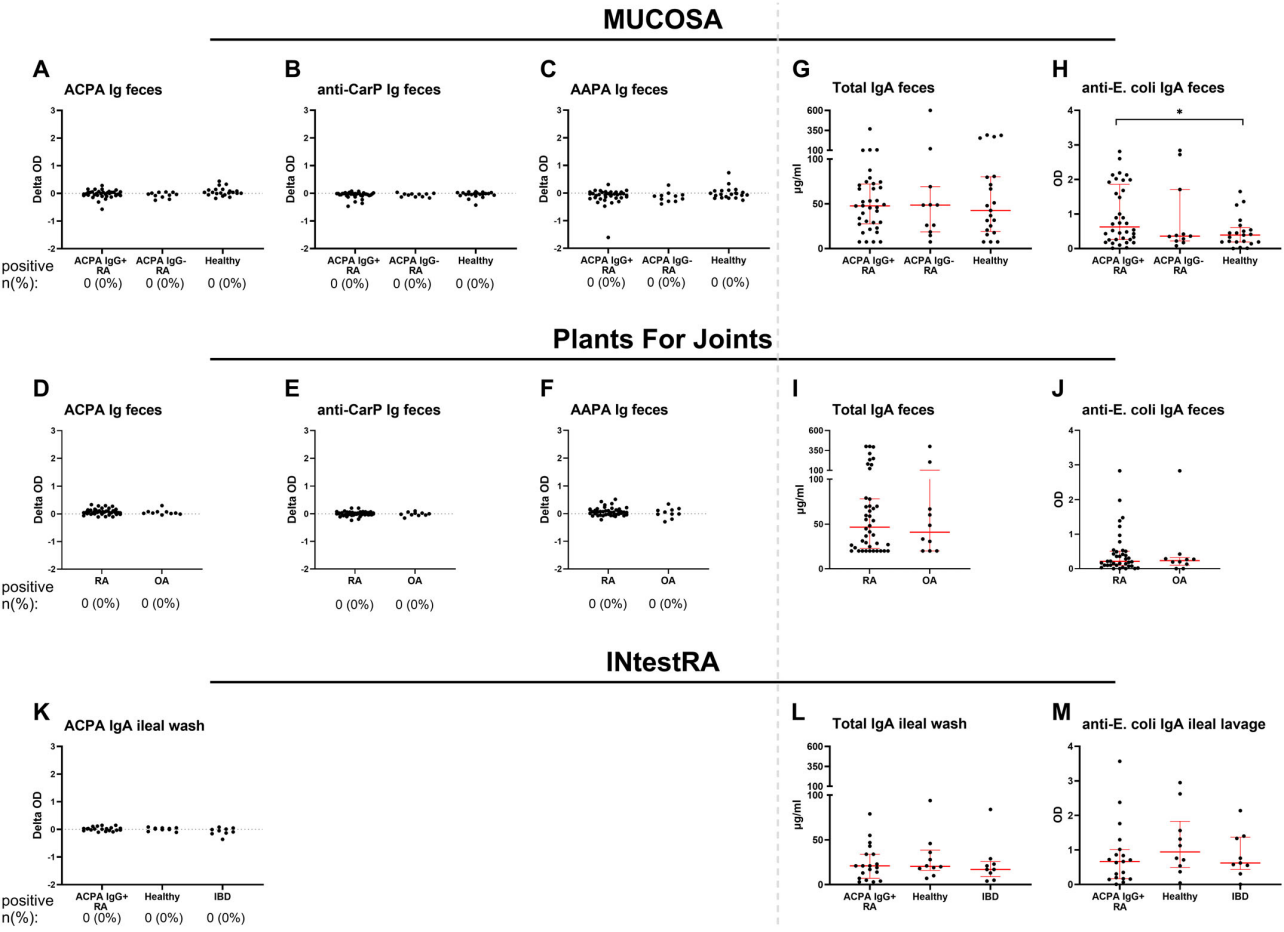
AMPA Ig in feces and ileal lavage of patients with RA. (A–C) ACPA, anti‐CarP, and AAPA Ig, respectively, in feces from patients and healthy donors in the MUCOSA study are shown. Groups on *x* axis are based on diagnosis and seropositivity. (D–F) AMPA Ig in feces of the Plants for Joints cohort is shown. (G–J) Total IgA levels in μg/mL (G and I) and OD (H and J) on the anti–*E. coli* IgA ELISA in the same feces samples. (K) ACPA IgA in ileal lavage samples from the IntestRA study is shown. (L) Total IgA levels and (M) anti–*E. coli* IgA OD in the same ileal lavage samples are shown. For AMPAs, the number (%) of positive patients is given. For AMPAs, the *y* axis depicts difference in OD (delta OD) between the modified and unmodified peptide. Red bars show the median and interquartile range. *P < 0.05. AAPA, anti–acetylated protein antibody; ACPA, anti–citrullinated protein antibody; AMPA, anti–modified protein antibody; CarP, carbamylated protein; ELISA, enzyme‐linked immunosorbent assay; IBD, inflammatory bowel disease; MUCOSA, MUCosal Origin of Serum Autoantibodies in rheumatoid arthritis; OA, osteoarthritis; OD, optical density; RA, rheumatoid arthritis. Color figure can be viewed in the online issue, which is available at http://onlinelibrary.wiley.com/doi/10.1002/art.43036/abstract.

**Figure 5:**
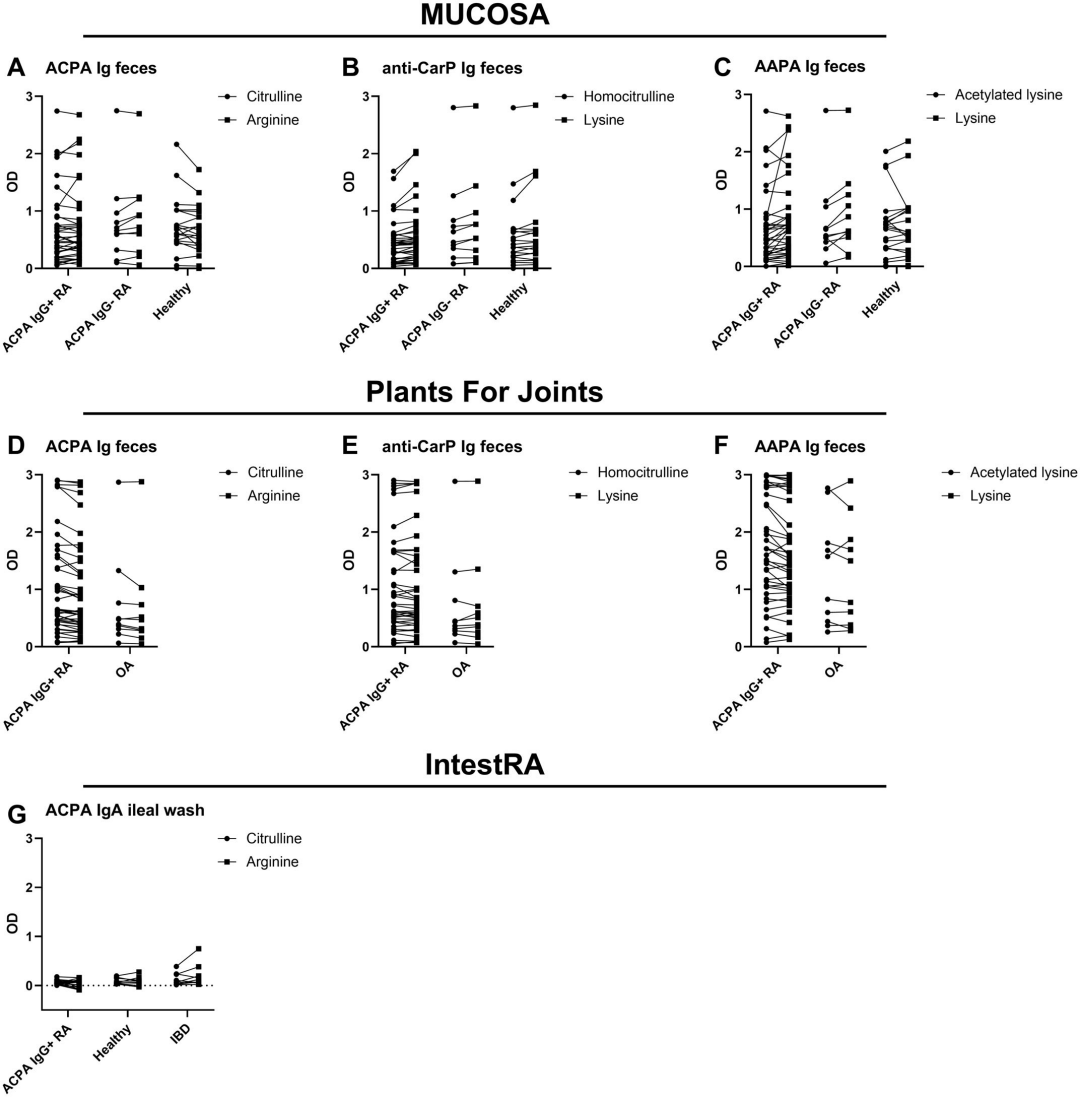
(A–G) Paired OD values on the modified and unmodified peptide for each AMPA Ig in feces of patients and controls in the MUCOSA study (A, C, E) and in the Plants for Joints trial (B, D, F) and ACPA IgA in ileal wash in patients and controls in the IntestRA study (G). AAPA, anti–acetylated protein antibody; ACPA, anti–citrullinated protein antibody; AMPA, anti–modified protein antibody; CarP, carbamylated protein; IBD, inflammatory bowel disease; MUCOSA, MUCosal Origin of Serum Autoantibodies in rheumatoid arthritis; OA, osteoarthritis; OD, optical density; RA, rheumatoid arthritis.

In the MUCOSA study, no ACPA, anti‐CarP, or AAPA were found in feces samples of patients with RA or healthy controls (Figure 4A–C), as there was almost no difference between the signals on the modified and unmodified peptides. Also, in the PFJ trial, no AMPA were found in feces (Figure 4D–F). Like in saliva, reactivity to the modified and unmodified peptides was evaluated separately in feces as well. Multiple ACPA‐seropositive patients with RA, ACPA‐seronegative patients with RA, and controls showed an OD >1 to the citrullinated peptide in their stool but also to unmodified arginine‐containing peptide, which was thus considered as nonspecific binding/AMPA negative (Figure 5A and D). Similar results were found for anti‐CarP and AAPA (Figure 5B,C,E,F) in both cohorts. The high OD signals on the unmodified peptides in feces are in contrast to saliva, for which only seropositive patients with RA showed a high background (Supplementary Figure 3).

To determine whether the fecal supernatants contained sufficient amounts of Ig to fall within the detection range of our ELISAs, total IgA levels were measured. Fecal supernatants in the MUCOSA study contained a median of 48 μg/mL total IgA, with no significant differences among seropositive patients with RA, seronegative patients with RA, and healthy donors (Figure 4G). Similar results were found in the PFJ trial (Figure 4I). These total IgA levels are roughly comparable with the amount of total IgA in the diluted saliva samples used for AMPA ELISA. Furthermore, anti–*E. coli* IgA was used as an additional (antigen‐specific) control. High anti–*E. coli* signals could both be detected in patients with RA and healthy donors. Interestingly, anti–*E. coli* reactivity was significantly higher in seropositive patients with RA compared with healthy donors in the MUCOSA study (*P* = 0.04) (Figure 4H), although the numbers are small. In the PFJ trial, no significant difference was observed (Figure 4J). These data indicate that the methods used are able to detect the presence of (antigen‐specific) antibodies in feces samples in general and that the lack of AMPA signal is not due to the absence of total IgA in these samples.

To substantiate our findings regarding the intestines, we also investigated ileal wash samples, collected via colonoscopy in the independent IntestRA cohort. Antibodies in such samples might be less prone to degradation compared with feces. However, also in the ileal wash samples, no ACPA IgA was detected (Figure 4K), whereas total IgA (median 20.5 μg/mL) (Figure 4L) and anti–*E. coli* IgA (Figure 4M) were detectable. ODs for both the modified and unmodified peptides were overall low (Figure 5G). Total IgA levels and anti–*E. coli* antibody signals were also slightly lower compared with the feces samples, possibly due to dilution by the lavage fluid instilled in the ileum used to collect these samples.

### Calprotectin in feces of patients with RA

Because previous reports have suggested that RA might be characterized by a leaky intestinal barrier, gut dysbiosis, and inflammation,^3^, ^11^, ^29^, ^30^ intestinal inflammation was investigated in the MUCOSA study. Calprotectin was measured in feces of both patients with RA and healthy donors by commercial ELISA (Orgentec) (Supplementary Figure 4A). Values above 200 μg/g are considered significantly elevated and reflect active inflammatory intestinal disease. Six out of 47 patients with RA and 1 out of 21 healthy controls had calprotectin values >200 μg/g, and an additional 7 patients had slightly elevated calprotectin levels between 100 and 200 μg/g. However, five of seven participants with strongly elevated calprotectin levels and four out of seven participants with slightly elevated calprotectin levels reported regular nonsteroidal anti‐inflammatory drug (NSAID) treatment (Supplementary Figure 4B) (ns, *P* = 0.07). NSAIDs are known to cause gastrointestinal mucosal damage,^31^ and it has been reported that two weeks of NSAID treatment in healthy individuals can lead to significantly elevated fecal calprotectin levels.^32^

## DISCUSSION

This study aimed to examine the RA‐associated autoantibody profile in various mucosal compartments. In the saliva of patients with established seropositive RA, we found that ACPA, anti‐CarP, and AAPA IgA antibodies can all be present, although in modest quantities compared with serum. Differences in the percentage of patients with ACPA‐positive saliva (17%–40% in ACPA IgG‐seropositive RA) among cohorts might reflect the inclusion of only patients with moderate to high ACPA serum levels in the IntestRA study. When salivary AMPA are present, the breadth of the response is similar to the AMPA serum profile. In contrast, no AMPA were found in feces of the same patients with RA, although the fecal supernatants did contain total IgA and anti–*E. coli* IgA antibodies. These findings were confirmed in feces and in ileal lavage samples from two independent cohorts of patients with RA. Our findings suggest that secretion of AMPA is limited to certain mucosal sites, with local secretion of all AMPA taking place in the oral cavity, but not to a detectable degree in the lower intestinal tract.

These observations are in line with previous studies showing the presence of salivary ACPA IgA in patients with RA.^17^ ACPA IgA and IgG were also previously found in sputum of seropositive patients with RA and first‐degree relatives (FDRs) of patients with RA.^16^ Some of the FDRs who were positive for ACPA in their sputum were serum ACPA negative, indicating that sputum ACPAs are produced locally and can precede or occur independently of a serological ACPA response. Our findings provide further evidence that autoantibodies found in mucosal secretions can be secreted locally at mucosal sites because the AMPA isotype present in saliva was not always measurable in serum. The low amount of IgG present in the saliva samples may further support that both the detected AMPAs and RF originated from the mucosal lining of the oral cavity instead of leaking from serum because leakage would have led to a higher quantity of IgG as the most abundant isotype present in serum. Furthermore, monomeric IgA from serum cannot be actively transported over the mucosal epithelium by the polymeric immunoglobulin receptor, whereas mucosal‐derived dimeric IgA can.

These findings raise the question of where the initial activation of autoreactive B cells in RA can take place and which triggers elicit these anti–modified protein responses. Activated B cells re‐enter the tissue where they were activated based on homing marker expression, although there probably is some crossover to other, often anatomically closely related tissues.^26^ This suggests that the cells secreting AMPA in the oral mucosa are probably derived from B cells activated in local lymphoid tissue. Our study shows that the salivary AMPA response not only includes ACPA, but also antibody responses against carbamylated and acetylated proteins, suggesting the local presence of these antigens. Interestingly, bacteria can acetylate self‐proteins^21^, ^22^ and thus might evoke an anti–acetylated bacterial protein response, which could be cross‐reactive to acetylated self‐proteins. It is hypothesized that, via this mechanism, the antibody responses against acetylated bacterial content can contribute to diversification and epitope spreading of the AMPA response in RA. Furthermore, in the MUCOSA study, seropositive patients with RA tended to have a higher reactivity in saliva toward the unmodified peptides compared with seronegative patients with RA and healthy donors, although this was not as clear in the IntestRA cohort. This higher reactivity toward unmodified peptides in seropositive patients with RA could also point to activated humoral immune responses in general in these patients, for example, due to decreased barrier function or local inflammation.

Our study did not include a dental examination to determine the presence of periodontitis (inflammation of the gums), which can be caused by bacterial infection. To gather some information on oral inflammation nonetheless, total protein content, MMP‐8 levels, and total IgA were measured in saliva. No association between these markers of inflammation and ACPA positivity in saliva was seen. This could be explained in several ways: The sensitivity of these markers might be more limited than a dental examination, and gingivitis or periodontitis could have been missed. Alternatively, a true lack of association could suggest that oral production of autoantibodies is independent of simultaneously occurring mucosal inflammation and would either not require inflammation at all or could be related to barrier dysfunction and inflammation in the past.

Not only the oral mucosa, but also the gut could represent a large source of citrullinated, carbamylated, and acetylated (microbial) proteins. From an immunologic point of view, it appears conceivable that a T cell response against post‐translationally modified bacteria (as foreign/nonself) may provide the required T cell help to activate self‐reactive AMPA‐directed B cells. Reactivity to intestinal bacteria was found to be a normal property of the human CD4+ T cell repertoire in healthy individuals.^33^ Moreover, it has been described that monoclonal ACPA derived from individuals at risk for RA can bind bacterial isolates from human feces.^20^ Based on these findings, one would have expected to find AMPAs in intestinal secretions as well, but we did not find ACPA, anti‐CarP, or AAPA in feces or ileal wash samples. This suggests that there is no substantial ACPA production in the lower intestinal tract. Anti–*E. coli* IgA and total IgA were measurable in these intestinal samples, indicating that the methodology is adequate to detect (antigen‐specific) antibodies.

Our results suggest that mucosal AMPA production is site specific, with local secretion of AMPA taking place in the oral mucosa, but not substantially in the gut. In addition, earlier studies provide evidence for AMPA responses in the airways.^16^, ^18^ This spatial variation might be due to differences in the local microenvironment, such as antigen availability, and local inflammatory processes, such as NETosis (release of Neutrophil Extracellular Traps). However, there are several other reasons why AMPA might not be detectable in the gut, for example, due to strong binding to their antigen, degradation of antibodies by digestive enzymes, or the amount of AMPA being under the detection limit of our assays. The facts that total IgA and anti–*E. coli* IgA were detectable in feces and that ACPA were also not present in ileal lavage samples, which might be less prone to degradation, make it more likely that the gut is not a major site of secretion for AMPA. Nevertheless, further research on barrier dysfunction and presence of PTMs in the intestinal tract of seropositive patients with RA is warranted because there are other potential mechanisms via which the intestinal mucosal compartment could contribute to the systemic AMPA response that are beyond the scope of our study, such as microbiome dysbiosis, decreased intestinal barrier function, and trafficking of immune cells primed in the intestine to the systemic circulation. For example, in one cohort, there was a difference in fecal anti–*E. coli* reactivity between seropositive patients with RA and healthy donors, which could point to increased interactions between gut bacteria and the immune system in RA.

Our study provides new insights in the autoantibody profile at mucosal surfaces, but it comes with some limitations. Most patients had longstanding RA and used various immunosuppressive therapies, which could have influenced the results. Previous studies investigating the effect of antirheumatic treatment on serologic AMPA responses have revealed that the presence of AMPA in serum is quite stable under treatment,^34^ but it is unknown whether mucosal AMPA responses originate from antibody‐secreting cells with similar (long‐lived) characteristics. Positivity for secretory ACPA in serum declined more strongly compared with ACPA IgG after initiation of therapy.^35^ Furthermore, despite the use of three independent cohorts to verify our findings, the number of patients included is limited. Because of the COVID‐19 pandemic, we were prohibited from collecting paired sputum samples in the MUCOSA study, as originally planned. Therefore, whether anti‐CarP and AAPA are also present in sputum of patients with RA remains unknown.

To the best of our knowledge, our study nonetheless represents the most extensive investigation to date of a large variety of autoantibodies in a diverse array of bodily fluids. Our results show that ACPA, anti‐CarP, and AAPA can be secreted locally in the oral mucosa. This suggests that local immune responses against PTMs, for example, in the context of an antibacterial response, might contribute to the development and diversification of the AMPA response in patients with RA. No support for local AMPA secretion in the lower intestinal tract was found. This study, therefore, for the first time sheds light on one of the possible roles (or potential lack thereof) of the intestinal mucosa in the onset of the AMPA responses in RA.

## AUTHOR CONTRIBUTIONS

All authors contributed to at least one of the following manuscript preparation roles: conceptualization AND/OR methodology, software, investigation, formal analysis, data curation, visualization, and validation AND drafting or reviewing/editing the final draft. As corresponding author, Dr van der Woude confirms that all authors have provided the final approval of the version to be published, and takes responsibility for the affirmations regarding article submission (eg, not under consideration by another journal), the integrity of the data presented, and the statements regarding compliance with institutional review board/Declaration of Helsinki requirements.

## Supporting information


Disclosure form

**Appendix S1:** Supplementary Information
