# Supplementary material for: Evidence of Site‐Specific Mucosal Autoantibody Secretion in Rheumatoid Arthritis
Source: Arthritis Rheumatol. 2024 Nov 18;77(3):272–82. doi: 10.1002/art.43036 (PMC11865693; doi:10.1002/art.43036)

Supplementary data 1: Methodology MUCOSA study and feces samples Plant For Joints

Patients and sample collection

All patients with a definite diagnosis of rheumatoid arthritis based on the ACR/EULAR 2010 criteria visiting the outpatient clinic of the Leiden University Medical Center (LUMC) were eligible for inclusion, regardless of disease duration or treatment. As a control, healthy donors without any history of inflammatory arthritis were included. All participants were 18 years or older and provided written consent. Individuals with current upper airway infection, presence of oral ulcers, dental treatment within the previous month or known IBD were excluded. Data on smoking and medication use was collected using a digital patient survey. Medication use was verified by status review. Participants were asked to refrain from vigorous exercise for 2 hours prior to saliva collection and refrain from food or beverage (except water), smoking, chewing gum and brushing their teeth for 1 hour prior. Before starting saliva collection, the participants were asked to rinse the mouth with water. Saliva was collected by ‘passive drooling’: the donor leaning forward allowing saliva to drip into a test tube for 10 to 15 minutes. Saliva samples were incubated at 56°C for 45 minutes as a precautionary measure to prevent spreading of SarS-CoV-2 virus particles. After incubation samples were homogenized and spun down at 7000g for 10 minutes. Supernatants were collected and stored at -80°C until further use. After thawing, saliva samples were spun down for 5 minutes at 5000g directly before use.

Feces was self-collected at home within 2 weeks of saliva and serum donation. The feces was immediately stored in a house-hold freezer for a maximum of 2 days, before it was transferred on dry ice to a -80 freezer. Saliva and feces samples containing macroscopic blood were excluded.

To prepare protein fractions of the feces sample to use on ELISA, circa 1 gram of feces was diluted in 5ml/gram feces dilution buffer (PBS + 0.05M EDTA + 1.66mM PMSF + 0.1mg/ml soybean trypsin inhibitor (sigma)). Glass beads (2mm, Merck) were added, and samples were mixed vigorously for 10-20 minutes until homogeneous and spun down at 2000g for 10 minutes at 4°C. Supernatants were collected and centrifuged for 10 minutes at 10.000g. Supernatants were aliquoted and stored at -20°C until further use. Feces supernatants were spun down for 5 minutes at 10000g directly before use. The same protocols for preparing the feces samples as well as for the ELISAs on fecal extracts were used in the Plant For Joints study.

AMPA measurements using ELISA

ELISAs were performed using 384 Well Flat Bottom High Bind Microplates (Corning), unless stated otherwise. For AMPA detection, biotinylated CCP2 (patent EP2071335) or similar peptides where the citrulline was replaced by a homocitrulline (CHcitP2) or acetylated lysine (CAcetylP2) were used. Control peptide containing an arginine (CargP2) instead of citrulline, or a lysine (ClysP2) instead of a homocitrulline or acetylated lysine were coated on the same plate. The peptides including the patent protected CCP2 and CargP2 were provided by Dr. J.W. Drijfhout (Dept. of IHB, LUMC). After each step, ELISA plates were washed with PBS/0.005% Tween 20. All ELISAs are visualized with ABTS/H_2_O_2_. For ACPA, anti-CarP and AAPA measurements in saliva and feces a modified version of the serum assay was used. The biotinylated modified or control peptides were coated in a concentration of 1 µg/ml in PBS/0.1%BSA on streptavidin coated plates (microcoat, standard capacity; 604500) for serum or on 384 well microplates pre-incubated overnight at 4°C with 1 μg/ml streptavidin (invitrogen) for saliva/feces and incubated for 1 hour at room temperature (RT). Serum was diluted 1:50 in PBS/0.05% Tween/ 1% BSA (PBT) (for IgG) or PBS/1%BSA/50 mM TRIS/0.05% Tween, pH 8.0 (PBTT) (for IgA) and incubated for 1 hour at 37°C (ACPA, anti-CarP) or overnight at 4°C (AAPA). Saliva was diluted 1:4 in PBS/0.05% Tween/2%Casein (PTC) and incubated for 2 hours at 37°C. Feces homogenates were added undiluted and incubated overnight at 4°C on ice. A pooled serum standard was used to calculate arbitrary units, and positive and negative serum controls were included on each plate. After washing, plates were incubated with rabbit anti-human-IgG-HRP (DAKO, P0214) 1:4000 (anti-CarP, AAPA) or 1:8000 (ACPA) in PBT for serum or goat anti-human-IgA-HRP (Invitrogen; A18781) 1:5000 for serum and 1:3000 for saliva in PBTT for 1 hour at 37°C. For feces, goat anti-human-Ig-HRP (Bethyl) 1:2000 in PBTT was added an incubated for 3.5h at 4°C on ice. Blanks were subtracted and samples were considered AMPA positive when they were above the cut-off and the OD (optical density) of the modified peptide was larger than 2 times the OD on the unmodified peptide. The cut-offs were determined based on the mean plus 2 times the standard deviation of the OD of healthy controls. In case the cut-off was below the linear range of the standard, the lowest point of the linear range was used as cut-off.

Rheumatoid factor ELISA

For RF IgM and IgA ELISA, ELISA plates (Nunc maxisorp plates (VWR) for serum) were incubated overnight at room temperature with 10 μg/ml human IgG for RF IgM (Jackson Immunoresearch; 009-000-003) or 5 μg/ml rabbit IgG for RF IgA (Nordic-MUbio) and blocked for 1 hour at 37°C with PBS/1%BSA (RF IgM) or PBS/2%Casein (RF IgA). Serum samples were diluted 1:100 in PBT for RF IgM and 1:185 in PBTT for RF IgA and incubated for 1 hour at 37°C. Saliva samples were diluted 1:4 in PTC and incubated for 2 hours at 37°C. A commercial standard (N/T Rheumatology control SL/2, Siemens) was used to calculate arbitrary units. Plates were incubated with goat anti-human-IgM-HRP (Millipore; AP114P) 0,3 μg/ml in PBT or goat anti-human-IgA-HRP 1:5000 in PBTT for 1 hour at 37°C.

Total IgA and total IgG ELISA

For total IgA level measurements in saliva and feces, ELISA plates were coated with goat anti-human-IgA-Fc (Bethyl; A80-102A) 10 μg/ml in bicarbonate/carbonate coating buffer (pH 9.6). All incubation steps were performed for 1 hour at RT, except blocking with PBT which was done for 30 minutes at RT. Two dilutions were used of both saliva and feces: saliva was diluted 1:400 and 1:800 and feces 1:80 or 1:100 and 1:400 in PBTT. A serial dilution of purified secretory IgA (Bio-rad PHP133, 1mg/ml) was used as standard. Goat anti-human-IgA-HRP (Bethyl; A80-102P), diluted to 50 ng/ml in PBTT, was used for IgA detection. Total IgG levels in saliva were measured in a similar fashion, using goat anti-human-IgG-Fc (Bethyl; A80-104) diluted 10 μg/ml in bicarbonate/carbonate coating buffer (pH 9.6) as coating antibody and goat anti-human-IgG-HRP (Bethyl; A80-104P), diluted to 50 ng/ml in PBTT as detection antibody. A serial dilution of human IgG (Jackson Immunoresearch; 009-000-003), starting with 0.25ug/ml was used as standard. Saliva samples were diluted 1:50 and 1:200 in PBTT.

Total protein and MMP-8 measurement in saliva

For total protein measurements in saliva, Pierce BCA protein Assay kit (Thermoscientific) was used in combination with Nunc maxisorp plates. Saliva samples were diluted 1:2 in PBS before use and measurements were performed conform manufacturer’s protocol. Total MMP-8 ELISA kit (R&D systems; DMP800B) was used to determine matrix metalloproteinase 8 levels (MMP-8) in saliva. The kit was used according to manufacturer’s instructions. Saliva samples were diluted 1:15 and a log-log standard curve was used to calculate MMP-8 levels.

Anti-E. coli and calprotectin ELISA

Escherichia coli (E. coli) of strain BL21 (kind gift of Can Amaran) were cultured locally manufactured M9 medium (containing KH_2_PO_4_ (3 g/L), Na_2_HPO_4_ (6 g/L), NaCl (0.5 g/L), MgSO_4_ (1 mmol/L), CaCl_2_ (0.1 mmol/L) and lysed using French press at 10,000 psi. The lysis procedure was performed three times. The solution with lysed bacteria was spun down at 21,000g, 4°C for 60 min, and the supernatant was collected. Concentration was measured with spectrophotometry. ELISA plates were coated with 10µg/ml E. coli lysate overnight at 4°C. After blocking with PBT for 1 hour at 37°C, undiluted fecal extracts and a pooled serum standard were added and incubated overnight at 4°C. Serum samples and purified secretory IgA were taken along as controls. Two HRP-labelled detection antibodies were used. First, goat anti-human-IgA-HRP was added and after washing, polyclonal rabbit anti-goat Ig-HRP (DAKO, P0449) was used. Both antibodies were diluted 1:1000 in PBTT and incubated for 1 hour at 37°C. Afterwards, signals were visualized with ABTS. Calprotectin in feces was measured using a commercial calprotectin ELISA kit (kindly provided by Orgentec; ORG580) according to manufacturer’s instruction.

Supplementary data 2: Methodology IntestRA study

Study subjects and samples

20 patients with RA (10 with disease duration < 1 year and 10 with established RA), 10 patients with Crohn’s disease and 10 healthy controls from the County of Dalarna, Sweden, were included between 2016 and 2019 in the IntestRA study. Only RA patients with a moderate or high level of IgG anti-CCP in serum were included, as these were expected to more likely have detectable ACPA in the intestine. Patients with known Crohn’s disease with engagement of terminal ileum and a scheduled colonoscopy were recruited consecutively from the endoscopy department’s waiting list. Indication for colonoscopy was assessment of disease activity. Persons participating in a screening study for colorectal cancer were asked to also contribute with samples from terminal ileum. None of these patients were diagnosed with colorectal cancer during the procedure.

Serum and saliva samples were collected at the rheumatology clinic. Participants were asked to restrain from eating, drinking other liquids than water, brushing teeth or smoking one hour before saliva sampling. Saliva was collected using passive secretion during 10 min, the sample was kept on ice and centrifuged 5 minutes at 5000 g. Serum samples were centrifuged 5 minutes at 5000g.

A colonoscopy was performed in all patients with at least 5 cm intubation of terminal ileum. 50 mL PBS was first instilled into the intestinal lumen using a catheter through the working channel of the endoscope. Intestinal fluid was then aspirated through the catheter. Ileal wash fluid samples were centrifuged 5 minutes at 5000 g, frozen within one hour after collection and stored at -80^o^C until further analyses.

IgA ACPA in saliva

Commercially available serum IgG-class anti-cyclic citrullinated peptide (anti-CCP) enzyme-linked immunoassays (ELISA) tests (CCPlus® Immunoscan, Svar Life Science) were modified to analyze IgA ACPA in saliva. All samples were analyzed in duplicate. Saliva samples were thawed at room temperature and spun down (11000g at 4^o^C for 10 minutes) directly before use, to remove non-soluble material. The supernatant was diluted to a final concentration of 1:20. The secondary antibody, polyclonal rabbit anti-human-IgA-HRP (DakoCytomation) was diluted 1:200. To adjust for non-specific IgA adsorption, all samples were tested against a control peptide (cyclic arginine peptide, CAP, EuroDiagnostica AB). Anti-CCP and anti-CAP analyses were performed in parallel and anti-CAP background levels were subtracted from anti-CCP values (delta optical density measurements).

IgA ACPA in ileal wash

Commercially available serum IgG anti-cyclic citrullinated peptide (anti-CCP) enzyme-linked immunoassays (ELISA) tests (CCPlus® Immunoscan, Svar Life Science) were modified to analyze IgA ACPA in ileal wash. All samples were analyzed in duplicate.

Ileal wash samples were thawed at room temperature and diluted to a final concentration of 1:5. Samples were incubated 1 hour at room temperature on the pre-coated 96-well plates. The secondary antibody, polyclonal rabbit anti-human-IgA-HRP (DakoCytomation) was diluted 1:200, added to the wells for 1h at RT. The plate was washed and the substrate Tetramethylbenzidine (TMB, Sigma Aldrich) added. The plate was read at 450nm. A positive control and blanks were included on the plate. Blanks were subtracted. To adjust for non-specific IgA adsorption, samples were tested against a control peptide (cyclic arginine peptide, CAP, EuroDiagnostica AB). Anti-CCP and anti-CAP analyses were performed in parallel and anti-CAP background levels were subtracted from anti-CCP values (delta optical density measurements). Samples were considered ACPA positive when they were above the cut-off determined based on the mean plus 2 times the standard deviation of the OD of healthy controls.

IgG and IgA ACPA in serum

Serum samples were analyzed for IgG ACPA according to the manufacturer’s instruction (CCPlus® Immunoscan, Svar Life Science), with cut-off set at 25 U/mL. IgA ACPA was analyzed using the same pre-coated CCP-plates, adding serum diluted 1:100. The secondary antibody of the kit was replaced with polyclonal rabbit anti-human-IgA-HRP (DakoCytomation) diluted 1:2000. A serum with high level of IgA ACPA was used to create a standard curve and cut-off levels for positive serum tests of IgA ACPA was set to 25 arbitrary units (AU)/mL.

### Total IgA in saliva and ileal wash samples

For total IgA level measurements in saliva and ileal wash, the IgA Saliva ELISA (IBL International, DM 59171) was performed according to the manufacturer’s instructions. Saliva was analyzed in dilution 1:1000 and Ileal wash in dilutions 1:100 and 1:500.

Anti-E. coli IgA in ileal wash samples

Anti-E. coli IgA in ileal washes samples was measured using the same in-house ELISA as used for the feces samples with minor modification. ELISA plates were coated with 10µg/ml E. coli lysate overnight at 4°C. After washing and blocking with PBT for 1 hour at RT, ileal washes, diluted 1:5, a positive control and a pooled serum standard were added and incubated overnight at 4°C. The plate was washed and goat anti-human-IgA-HRP (Invitrogen), diluted 1:1000 in PBTT, was added. Following incubation for 1 hour at 37°C the plate was washed and the substrate, TMB, added. The plate was read at 450nm (TECAN).

Supplementary Figure 1: AMPA positivity in saliva related to total IgA levels (µg/ml) in these saliva samples, collected in the MUCOSA study. Patients were divided into groups based on both saliva AMPA positivity and ACPA-seropositivity. The first column shows seropositive RA patients positive for any AMPA in saliva. Color coding is used to indicate for which AMPA they were positive in saliva; Orange: saliva ACPA single-positive, anti-CarP negative and AAPA negative patients, green: saliva ACPA negative, anti-CarP and AAPA double-positive patient, purple: saliva ACPA, anti-CarP and AAPA triple-positive patients. Error bar shows the median.


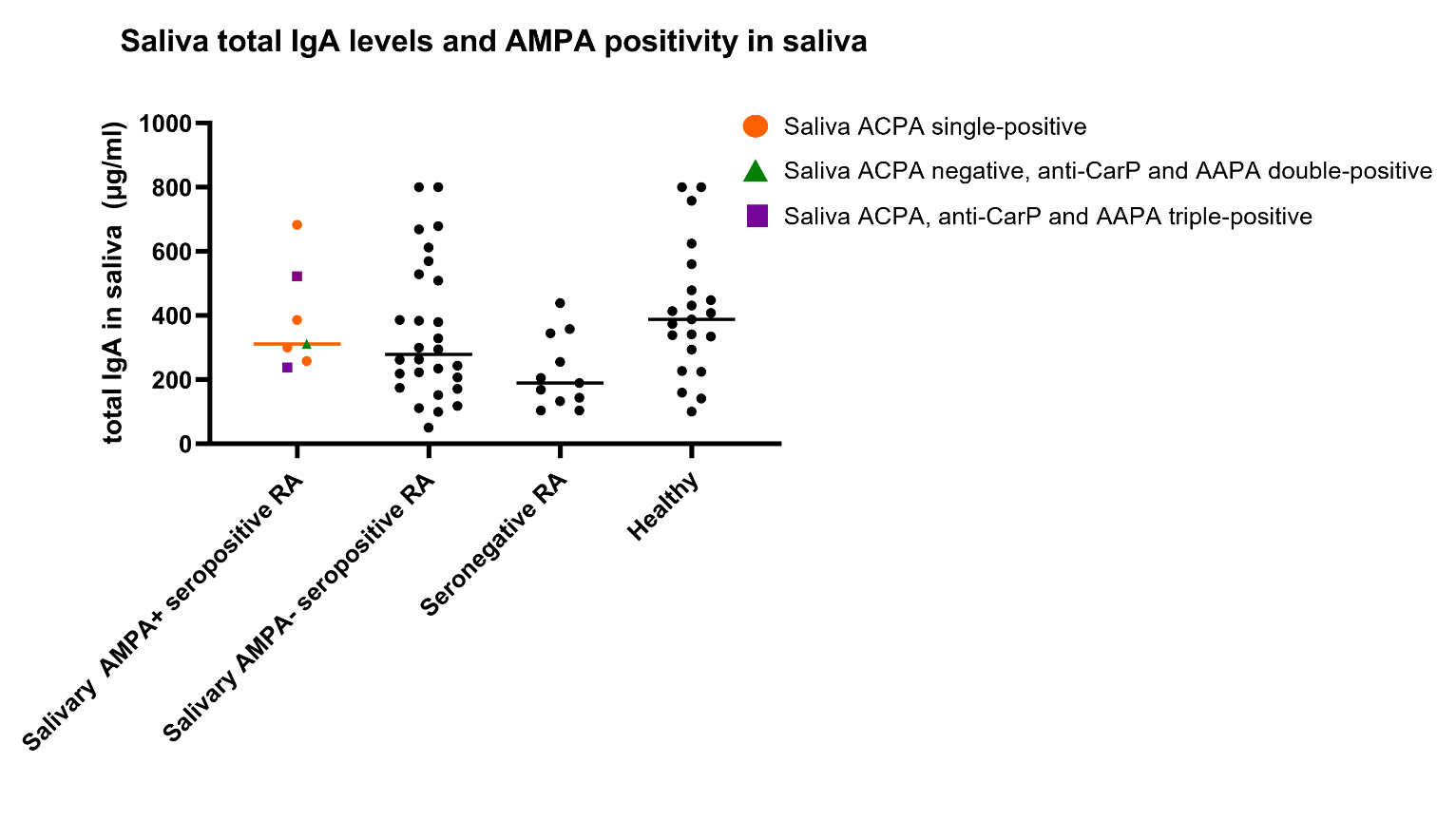


Supplementary figure 2: Inflammatory markers in saliva of patients in the MUCOSA study. Saliva-ACPA IgA positive RA patients, saliva-ACPA IgA negative RA patients and healthy donors are compared (A-C). Similar analyses were performed for ACPA seropositive RA patients, seronegative RA patients and healthy donors (D-F). The inflammatory markers measured in saliva are total protein levels, metalloproteinase-8 levels (MMP-8) and the amount of total IgA present in saliva. Mann-Whitney U tests were used to compare levels between groups. Red bars depict the median, *p<0.05.


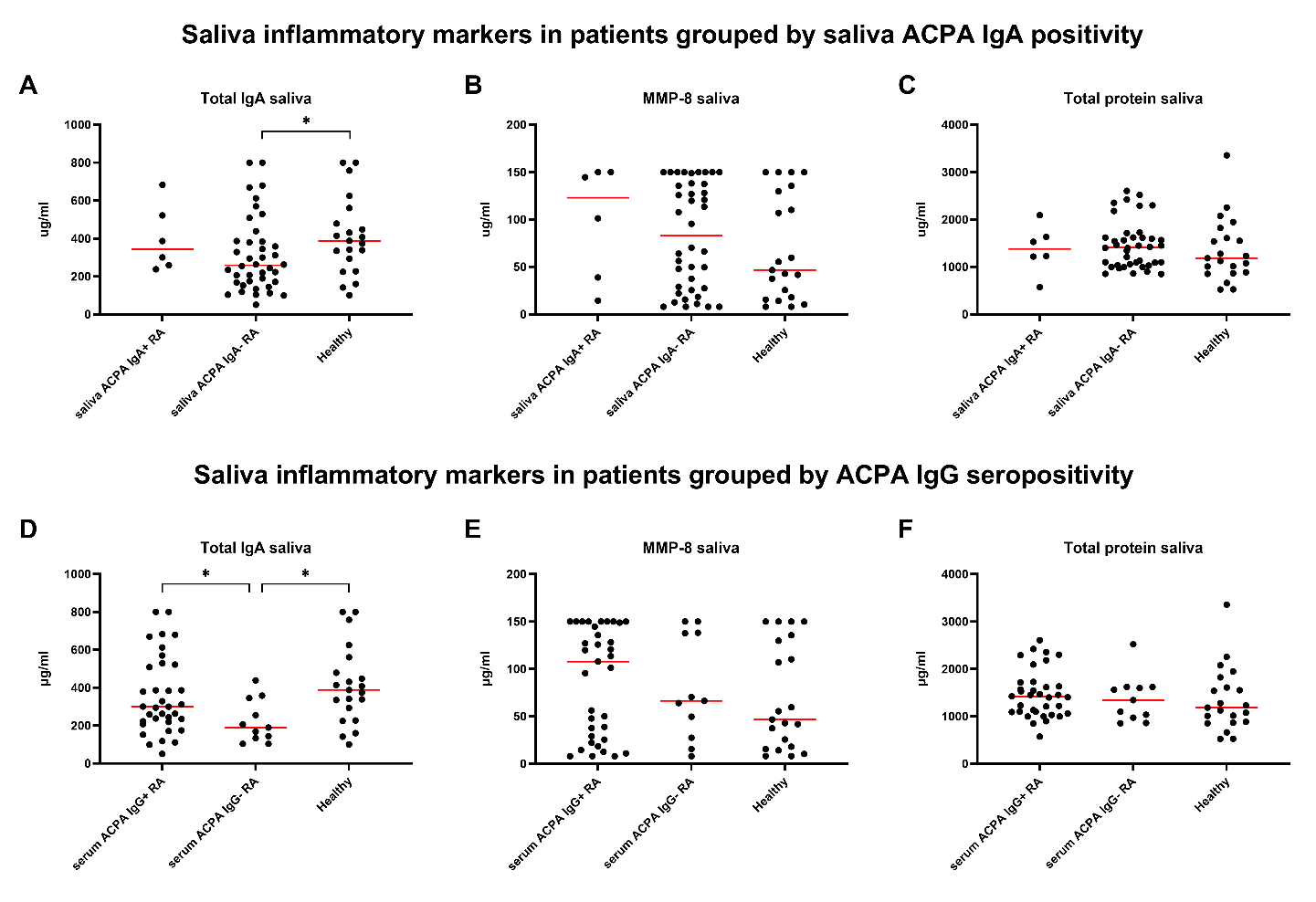


Supplementary table 1: The association between serum and saliva autoantibody positivity and smoking in RA patients in the MUCOSA study. Significance was tested with chi-square test or 2-sided Fisher’s exact test (indicated with *). Smoking was defined as ever- vs never-smokers.

| MUCOSA study | Never Smoked  n=21 | Ever Smoked  n=26 | p-value |
| --- | --- | --- | --- |
| ACPA IgG serum positive, n (%) | 14 (67) | 22 (85) | 0.15 |
| ACPA IgA serum positive, n (%) | 8 (38) | 10 (39) | 0.98 |
| RF IgM serum positive, n (%) | 12 (57) | 22 (85) | **0.04** |
| RF IgA serum positive, n (%) | 5 (24) | 16 (62) | **0.01** |
|  |  |  |  |
| ACPA IgA saliva positive, n (%) | 4 (19) | 2 (8) (n=25) | 0.39* |
| RF IgA saliva positive, n (%) | 10 (48) | 8 (32) (n=25) | 0.28 |

Supplementary figure 3: Paired signals on the modified and unmodified peptide for each AMPA in feces, saliva and serum collected in the MUCOSA study. A-C) Feces: The first group in each figure shows ACPA IgG seropositive RA patients, the middle group shows ACPA IgG seronegative RA patients. D-F) Saliva: The first group in each figure shows ACPA IgG seropositive RA patient positive for that AMPA IgA in saliva, the second group shows ACPA IgG seropositive RA patient who are negative for that AMPA IgA in saliva, the third group shows ACPA IgG seronegative RA patients. G-H) The first group shows RA patients positive for that specific AMPA IgA in serum, the second shows RA patients negative for that specific AMPA IgA in serum. The last column of all figures includes healthy donors.


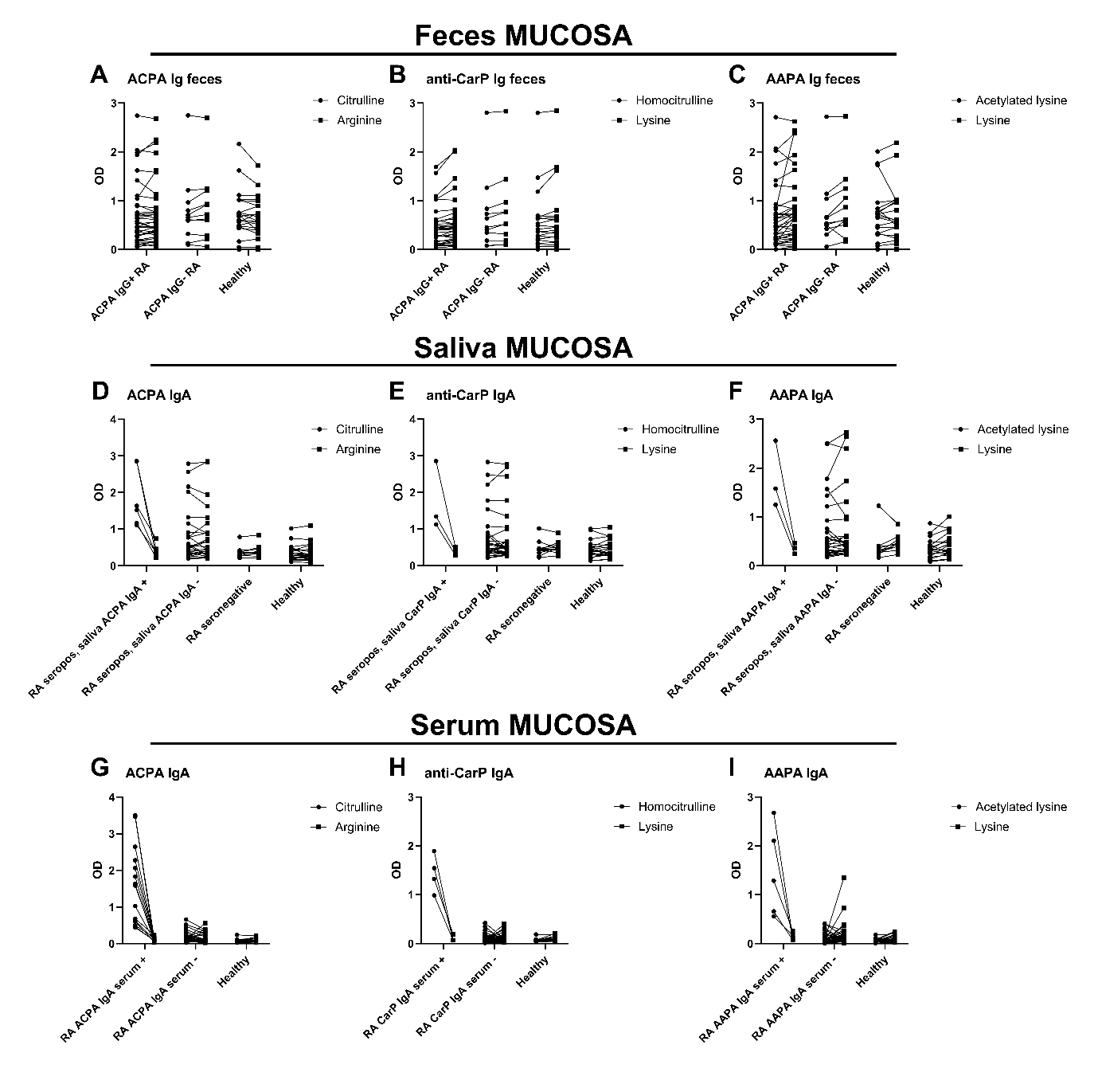


Supplementary figure 4: Calprotectin levels in feces samples collected in the MUCOSA study. A) Seropositive RA patients, seronegative RA patients and healthy donors and B) RA patients with and without NSAID use are compared. Calprotectin levels are measured by ELISA. Values above 200 µg/g are considered to be significantly elevated. NSAID used was based on the medication list in the electronic medical file and/or self-reported use as indicated in the patient survey. All NSAIDs (no information on dosage or frequency available) except for low dose acetylsalicylic acid as platelet aggregation inhibitor, was included in the NSAID group. Mann-Whitney U tests were used to compare levels between groups. Red bars depict median with interquartile range. *p<0.05


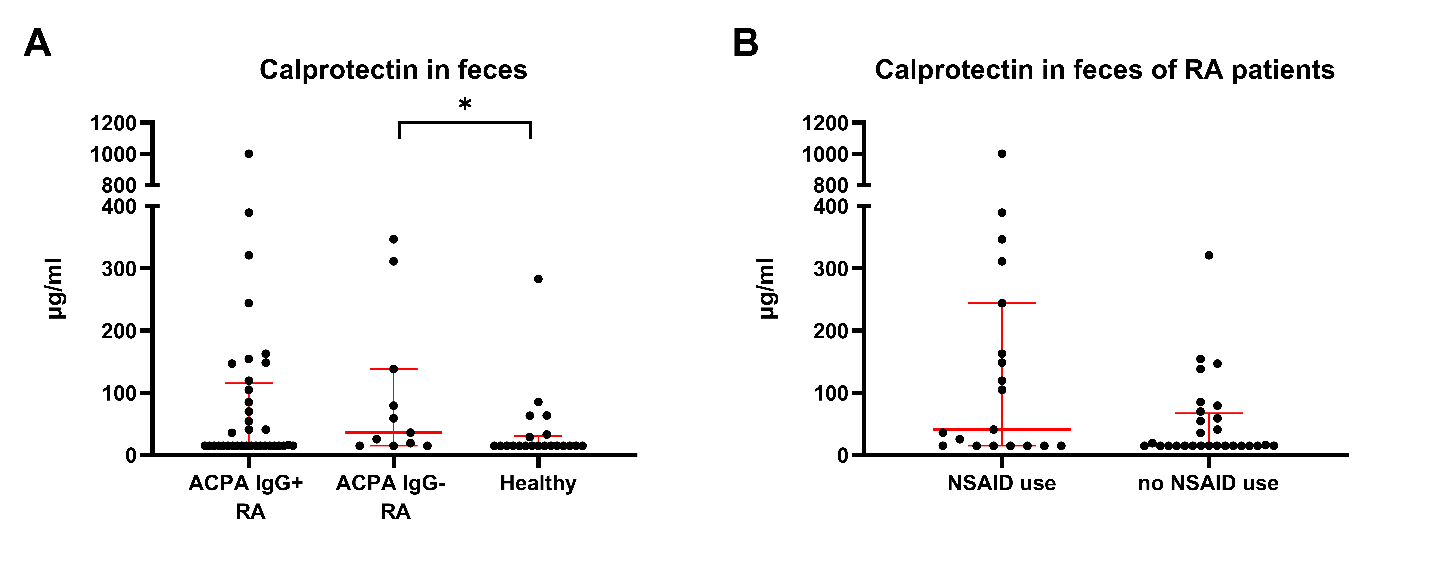

Supplement: Supplementary file 2 — Appendix S1: Supplementary Information [file ART-77-272-s002.docx]
